# Evaluating the Quality of the Core Entrustable Professional Activities for New Pharmacy Graduates

**DOI:** 10.3390/pharmacy11040126

**Published:** 2023-08-08

**Authors:** Abigail T. Elmes, Alan Schwartz, Ara Tekian, Jennie B. Jarrett

**Affiliations:** 1 Department of Pharmacy Practice, University of Illinois Chicago College of Pharmacy, Chicago, IL 60612, USA; jarrett8@uic.edu; 2 Department of Medical Education, University of Illinois Chicago College of Medicine, Chicago, IL 60612, USA; alansz@uic.edu (A.S.); tekian@uic.edu (A.T.); 3 American Medical Association, Chicago, IL 60611, USA

**Keywords:** entrustable professional activity, quality assessment, EQual rubric

## Abstract

This study aimed to evaluate the quality of the American Association of Colleges of Pharmacy Core Entrustable Professional Activities (Core EPAs) for New Pharmacy Graduates according to standards outlined in competency-based education literature utilizing the Queen’s EPA Quality (EQual) rubric. A cohort of pharmacists with EPA expertise rated Core EPA quality with the EQual rubric and provided recommendations for revisions. A generalizability study determined the reliability of the EQual ratings with pharmacist users. Nine pharmacists responded (4.4%). Most EPAs (9/15) did not reach the overall cut-off score, indicating low quality. EPAs 1 through 5 and EPA 14 (fulfill a medication order) were deemed high quality. EPA 12 (use evidence-based information to advance patient care) scored the lowest at 3.47 (SEM 0.29). EPA 14 scored the highest at 4.60 (SEM 0.14). EPA 15 (create a written plan for continuous professional development) was the only EPA to fail to reach the cut-off across all EQual domains. EPAs in the Patient Care Provider Domain received significantly higher ratings than other EPAs. On average, three respondents recommended revision for each. Most comments aligned with the EPA’s EQual rubric performance. The generalizability study analysis revealed excellent reliability (G = 0.80). Determining EPA quality utilizing objective measurement tools should drive EPA development and revisions to more accurately reflect the roles, responsibilities, and expectations of pharmacists on the healthcare team.

## 1. Introduction

Competency-based education (CBE) is a time-variable, learner-driven, outcomes-focused educational model that describes cognitive and non-cognitive components of knowledge, skills, and attitudes to be mastered across a continuous spectrum of learning [1,2]. However, learner assessments within CBE can be abstract and difficult to measure. Introduced to make assessments more concrete, entrustable professional activities (EPAs) describe tasks that can be entrusted to a learner with varying levels of supervision across their training [3]. By providing a framework outlining professional abilities, EPAs may be used to ensure graduates are “practice-ready” through demonstration of specific competencies, rather than assuming those competencies have been met by the end of the degree program [3]. To be considered as an EPA, a task should be a discrete unit of work, meaning that it has a clearly defined beginning and end [3,4,5]. It should be measurable, observable, and able to be performed independently [3]. Additionally, the task should be specifically entrusted to individuals within a profession and require training to complete [3,4].

In a move to parallel similar curricular transformations for CBE implementation in medical education, the American Association of Colleges of Pharmacy (AACP) utilized wide stakeholder input and consensus groups to create and disseminate 15 Core Entrustable Professional Activities for New Pharmacy Graduates (Core EPAs) in 2016 (Table 1) [6].

The Core EPAs have been integrated into pharmacy education nationwide within didactic and experiential curricula [7,8,9,10,11]. Despite having been previously critically appraised for their relevance to the profession [12,13,14,15,16], their quality and structure have not been objectively evaluated to ensure they meet specific characteristics related to EPA quality.

In combination with entrustment-supervision scales, EPAs provide a blueprint for faculty, preceptors, and students to standardize assessment expectations and set goals for progression across the curriculum. Additionally, a set of EPAs demonstrates and communicates a profession’s roles and responsibilities to members of that profession (e.g., pharmacists, pharmacy students, pharmacy organizations, etc.) and those who interact with them (e.g., other members of the healthcare team, patients, public, media, governmental agencies, policymakers, etc.) [17]. Finally, changes to a profession’s set of EPAs convey an evolution of their role to these stakeholders [17]. EPAs’ multidimensionality makes adherence to quality measures vital for valid and reliable learner assessment and professional advocacy efforts [17].

The Queen’s EPA Quality (EQual) rubric was produced and validated to objectively measure EPA quality [18]. The EQual rubric consists of 14 items utilizing 5-point criterion-based scales with descriptive anchors measuring three domains essential to EPAs: discrete units of work, entrustable and essential tasks of the profession, and having a curricular role. The EQual rubric defines overall cut-off scores and domain-specific cut-off scores to determine EPA quality and offers free electronic rater training resources [18,19].

Health professions outside of pharmacy have previously assessed EPA quality utilizing the EQual rubric [19,20]. In the final stages of EPA development, nursing educators utilized the EQual rubric to ensure the EPAs’ realism and generalizability [20]. Meyer and colleagues used six content experts to apply the EQual rubric to the Association of American Medical Colleges (AAMC) 13 Core EPAs for Entering Residency [19]. Overall, four AAMC EPAs were deemed unacceptable and three did not meet criteria in at least two of the EQual rubric’s three domains. This study highlighted the need for EPA quality evaluation before incorporating them into curricula [19].

The AACP’s Core EPAs’ pertinence and applicability to pharmacy practice has been established [13,14,15,16]. Haines and colleagues demonstrated the relevance of the Core EPAs using a questionnaire. While it is important to ensure that real-world pharmacists perform the EPAs in practice, the quality and structure of the Core EPAs should align with literature-based educational standards given their complex role across the pharmacy profession [17]. Notably, the 2021–2022 AACP Academic Affairs Committee revised the Core EPAs in the fall of 2022, by seeking feedback from various groups including AACP members and taskforces via online surveys, targeted interviews, and virtual and in-person town halls at national meetings [21]. Much of the public discussion focused on efficiency and streamlining with accreditation standards and learning outcomes rather than adhering to validated evidence-based frameworks such as nesting EPAs or using technology for assessment [4,22,23]. Impartial quality assessment of the Core EPAs was not presented in their work or demonstrated in the feedback [21]. Ultimately, the Committee changed EPA wording and reduced the number of EPAs from 15 to 13 statements [21]. This study was performed externally and separate from the Committee’s revision process.

The overall objective of this work is to enhance the Core EPAs for improved learner assessment in pharmacy education. The primary goal of this research is to evaluate the quality of the Core EPAs according to the standards outlined in the CBE literature with the EQual rubric as a framework for assessment. Despite being utilized in EPA quality assessment in other health professions, there is a need to ensure reliability of the EQual rubric in this novel application. A secondary aim is to confirm the interrater reliability of the EQual rubric with pharmacist users and pharmacy EPAs.

## 2. Materials and Methods

This prospective cohort study using survey methods utilized a cohort of nationally recognized EPA experts in the pharmacy field to rate the quality of the Core EPAs with the EQual rubric and provide recommendations for revisions. Methodology was built upon the framework employed by Meyer and colleagues to evaluate the AAMC Core EPAs for Entering Residency [19]. We hypothesized that Core EPAs in the Patient Care Provider domain (EPAs 1–5) are more likely to meet acceptability criteria versus EPAs 6 through 15.

A multi-modal purposive sampling approach was utilized to ensure all participants had adequate knowledge and experience with EPAs. Credibility of expertise was determined via EPA-related national organization committee selection and/or peer-reviewed publications. Members of the 2015–2016 AACP Academic Affairs Committee, who originally developed the Core EPAs, and the 2021–2022 committee, who were revising the Core EPAs at the time of data collection, were invited to participate. In a snowball sampling fashion, those committee members were asked to provide names of other pharmacists with intimate knowledge and experience with EPAs. Those mentioned names were asked to identify other pharmacists with EPA expertise, and names mentioned more than once in this process were invited to participate. Finally, pharmacists with EPA expertise were identified through authorship of at least two EPA-related pharmacy publications (identified via PubMed search) or at least one EPA-related presentation at an AACP Annual Meeting since 2018 (identified via past meeting programming materials). Investigators in this study that were identified via the sampling approach were excluded from the cohort. The goal was for a minimum of 10 experts to complete the EQual evaluation. All identified experts were invited via email. Those who completed the full evaluation tool were offered the opportunity to enter a raffle to win one of fifteen USD 100 Amazon gift cards for their time participating.

A 15 min pharmacy-centric training video was produced, vetted by experts, and published online for orientation and training of participants to the EQual rubric. Data were collected via the electronic survey instrument Qualtrics^®^ Software (Qualtrics 2018, Seattle, WA, USA). The first survey page included a consent form, the second page collected participant demographics, the third included the training video, and each subsequent page included one Core EPA next to the EQual rubric. For EQual rubric item 6, “This EPA is clearly distinguished from other EPAs in the framework”, the complete list of 15 Core EPAs was available for the participant to reference. For EQual rubric items 10 and 11, the word “physician” was changed to “pharmacist.” For example, the 5-rating under item 10 “Clearly expected of a physician as part of delivering competent clinical care” was changed to “Clearly expected of a pharmacist as part of delivering competent clinical care.” After participants rated each Core EPA, three prompts asked if and why the EPA requires revision along with a free-text opportunity to respond and recommend revisions. The Core EPAs were grouped within their respective domains, but the domains were presented to each participant in a random order to reduce bias.

The primary outcome was the number of Core EPAs assessed as high-quality, defined as exceeding the overall acceptability cut-off score (4.07). Secondary outcomes included each Core EPA’s domain-specific EQual score, whether it exceeds the domain-specific acceptability cut-off score, expert recommendations for revisions, and the interrater reliability of the EQual rubric with pharmacist users. Participants initially had three weeks to complete the evaluation, with the opportunity to extend to four weeks total, if needed.

Descriptive statistics were calculated for the overall and domain-specific EQual rubric scores for each Core EPA. Free text responses were summarized by investigators and organized into themes (e.g., overlaps with another EPA, addresses numerous tasks within one EPA, not measurable, etc.). We compared the scores for the Patient Care Provider domain EPAs with the other EPAs by fitting a linear mixed-effects model with a dichotomous predictor for Patient Care Provider (vs. other) domain and random effects of rater and EPA. We examined the association between each EPA’s performance on the EQual rubric relative to the cut-off (high/low quality) and whether the respondent recommended revision (yes/no) by applying McNemar’s test, and the overall association across EPAs by fitting a mixed-effects logistic regression model with random effects of rater and EPA. A generalizability study was performed to determine the reliability of the EQual ratings with EPA, rater, and EQual rubric item in a fully crossed design. Statistical analysis was performed using R 4.2 (R Core Team, Vienna, Austria). This study was deemed exempt by the University of Illinois Chicago Institutional Review Board.

## 3. Results

The sampling approach identified a total of 205 pharmacist EPA experts. Most respondents were identified by their EPA-related peer-reviewed publication record. Of the 205 pharmacists identified, nine completed the entire evaluation (4.4%), which included one member of each of the AACP Academic Affairs committees.

Most EPAs (9/15) did not score high enough on their overall EQual score to exceed the overall cut-off score of 4.07 (Table 2). The overall EQual score for EPAs 1 through 5 (Patient Care Provider Domain) and EPA 14 (fulfill a medication order) exceeded 4.07, indicating high quality. The lowest scoring EPA was EPA 12 (use evidence-based information to advance patient care) at 3.47 (SEM 0.29). The highest overall EQual score was EPA 14 at 4.60 (SEM 0.14). EPAs in the Patient Care Provider Domain received significantly higher ratings than other EPAs (mean difference = 0.64, 95% CI 0.32–0.96, *p* < 0.001).

Regarding domain-specific EQual scores, 11 EPAs (4 through 13 and 15) did not reach the cut-off for EQual Domain 1 (discrete units of work). The lowest scoring EPA within this domain was EPA 12 (use evidence-based information to advance patient care) at 2.68 and the highest scoring EPA was EPA 1 (collect information to identify a patient’s medication-related problems and health-related needs) at 4.52. Five EPAs (6, 7, 12, 13, and 15) did not reach the cut-off for EQual Domain 2 (entrustable tasks of the profession). The lowest scoring EPA within this domain was EPA 15 (create a written plan for continuous professional development) at 2.92 and the highest scoring EPA was EPA 14 (fulfill a medication order) at 4.78. Not only was EPA 15 the only Core EPA to not reach the cut-off for EQual Domain 3 (curricular role) at 3.28, but it was also the only Core EPA to fail to reach the cut-off across all EQual Domains.

The generalizability study analysis revealed excellent reliability (G = 0.80) based on nine reviewers rating an EPA. Table 3 presents the variance components. Most variance was associated with the EPAs themselves rather than the rater or the EQual rubric item. A follow-up multivariate generalizability study estimated the nine raters’ reliabilities at 0.80, 0.87, and 0.70 for EQual domain scores for domains 1, 2, and 3, respectively.

At least one respondent recommended revision for each EPA with an average of three respondents per EPA. The EPA with the most respondents recommending revision was EPA 12 (*n* = 5, 56%). No individual EPA’s McNemar test identified a significant association between whether that EPA reached the overall cut-off score (yes/no) and if the respondent recommended revision. However, the overall test for association across all EPAs did find that those rated under threshold were significantly more likely to have revisions recommended (adjusted odds ratio = 15.5, 95% CI 4.3–55.1, *p* < 0.001). In other words, the respondents were more likely to recommend revising the EPA when the EPA scored poorly on the EQual rubric.

Comments and recommendations for EPA revisions are summarized in Table 4. Most comments aligned with how the EPA performed on the EQual rubric. Notably, respondents recommended that EPAs 1–5, which were deemed to have acceptable quality by the EQual rubric, should all be separated into multiple EPAs. Other comments included rewording an EPA to address an area that performed poorly on the EQual rubric (observability, measurability, etc.) or eliminating it altogether, particularly if the EPA was not exclusive to the pharmacy profession.

## 4. Discussion

This research investigated the quality of the Core EPAs for New Pharmacy Graduates developed by AACP in 2016 utilizing the EQual rubric as an evaluation tool for EPA quality. The Patient Care Provider Domain was the only Core EPA Domain to meet overall quality scores for all EPAs within, supporting our hypothesis. This domain aligns with the Pharmacist’s Patient Care Process [24], a framework for a team-based, patient-centered comprehensive approach to pharmacy practice across all patient care settings that has been incorporated into accreditation standards for pharmacy education [25,26].

For the majority of EPAs that did not reach acceptable quality scores, the score was driven by low scores in EQual Domain 1 which outlines an EPA as a discrete unit of work. Domain 1 measures whether a task has a defined beginning and end and is specific, focused, observable, measurable, independently executable, and well-differentiated from other EPAs [18]. Most free text responses also aligned with this Domain as many comments and recommendations for revision indicated that the EPA overlapped with another within the set and that the EPA should be reworded for observability, measurability, etc. This suggests that EPAs that meet cut-off scores in other EQual Domains but fail to reach cut-off scores in Domain 1 may benefit from revising the wording of the task, rather than changing the task itself.

Numerous respondents recommended that some EPAs be separated into multiple EPAs for clarity, despite some of those EPAs reaching acceptable quality (see EPAs 1 through 5). This, coupled with the recommendation that the number of total EPAs for a training program range from 20 to 34 activities [27], indicates a need to expand the number of EPAs for use in pharmacy education. However, this need for expansion is in opposition with the direction of the 2022 Core EPA revisions (renamed to Curricular Outcomes and Entrustable Professional Activities (COEPA) for Pharmacy Graduates) which reduced the number of EPAs from 15 to 13 [21,28]. In these revisions, all Core EPAs, except for EPA 14 (fulfill a medication order), were either reworded in some capacity or removed altogether. In alignment with the results of this study, EPA 15, the only EPA to fail to reach acceptability cut-offs across all three EQual domains, was removed, along with EPAs 9 and 10.

The revisions also separated EPA 11 (Educate patients and professional colleagues regarding the appropriate use of medications) into two statements (“Educate the patient and others trusted by the patient regarding the appropriate use of a medication, device to administer a medication, or self-monitoring test” and “Deliver medication or health-related education to health professionals or the public”). While this change does not directly contradict the results of this study, our results indicate that EPA 11 overlapped with other clinical care activities and should have been reworded for focus.

Rather than expanding the EPAs that address numerous tasks into more than one activity as recommended by the respondents in this work, the Committee removed or reworded only one of the tasks within the activity. For example, EPA 3 (establish patient-centered goals and create a care plan for a patient in collaboration with the patient, caregiver(s), and other health professionals that is evidence-based and cost-effective) changed to “Create a care plan in collaboration with the patient, others trusted by the patient, and other health professionals to optimize pharmacologic and nonpharmacologic treatment” [21,28].

An example of other adjustments includes the revisions to EPA 8 (minimize adverse drug events and medication errors) which changed to “Report adverse drug events and/or medication errors in accordance with site specific protocols”. It is unclear whether any of the changes made by the Committee to the EPAs would improve their performance on the EQual rubric. As CBE is considered by the pharmacy Academy [29], there is a clear need to incorporate quality assessment processes into all steps of EPA revisions [30]. By reducing the number of EPAs or altering the EPAs, the Committee has limited the scope of practice in pharmacy and thereby the ability to assess practice-readiness across the profession and communicate the diverse roles and responsibilities of a pharmacist to a broad range of stakeholders [17].

A limitation of the study is that only nine raters participated despite a financial incentive. Although this number is greater than the number used in other studies applying the EQual rubric to evaluate EPA quality (e.g., six raters were used to evaluate EPA quality in medicine) [19], we had hoped to recruit considerably more raters. Potentially, members of the 2021–2022 AACP Academic Affairs Committee refrained from participating in the research due to a self-perceived conflict of interest as they were in the process of revising the Core EPAs at the time of data collection. Fortunately, this study demonstrated the interrater reliability of the EQual rubric in this context was sufficiently high to warrant the use of nine raters. With the interrater reliability established with pharmacist users, future research should explore quality assessment of the 2022 COEPAs for Pharmacy Graduates [28] and the integration of quality assessment processes in EPA development and revisions.

## 5. Conclusions

The majority of the Core EPAs were deemed to be of insufficient quality and require revision to be of high quality. This study establishes a process for revising pharmacy EPAs to improve their quality and align with evidence-based educational models in other health professions. Determination of EPA quality utilizing objective measurement tools like the EQual rubric should drive EPA development and revisions to more accurately reflect the current and evolving roles, responsibilities, and expectations of the pharmacist on a healthcare team [17].

## Figures and Tables

**Table 1:** The 2016 AACP Core Entrustable Professional Activities for New Pharmacy Graduates [6].

Domain	Entrustable Professional Activity
Patient Care Provider	Collect information to identify a patient’s medication-related problems and health-related needs. Analyze information to determine the effects of medication therapy, identify medication-related problems, and prioritize health-related needs. Establish patient-centered goals and create a care plan for a patient in collaboration with the patient, caregiver(s), and other health professionals that is evidence-based and cost-effective. Implement a care plan in collaboration with the patient, caregivers, and other health professionals. Follow-up and monitor a care plan.
Interprofessional Team Member	6. Collaborate as a member of an interprofessional team.
Population Health Promoter	7. Identify patients at risk for prevalent diseases in a population. 8. Minimize adverse drug events and medication errors. 9. Maximize the appropriate use of medications in a population. 10. Ensure that patients have been immunized against vaccine-preventable diseases.
Information Master	11. Educate patients and professional colleagues regarding the appropriate use of medications. 12. Use evidence-based information to advance patient care.
Practice Manager	13. Oversee the pharmacy operations for an assigned work shift. 14. Fulfill a medication order.
Self-Developer	15. Create a written plan for continuous professional development.

**Table 2:** Average EQual rubric scores of the Core EPAs for new pharmacy graduates (*n* = 9).

	Average EQual Rubric Score						No. to Recommend Revision
				Overall			
Core EPA	Domain 1 (4.17) *	Domain 2 (4.00) *	Domain 3 (4.00) *	Score (4.07) *	SEM	Range	
1	4.52	4.56	4.50	4.52	0.16	3.36–4.93	1
2	4.28	4.69	4.75	4.53	0.11	4.00–5.00	3
3	4.22	4.75	4.72	4.52	0.12	4.07–5.00	2
4	**4.00**	4.64	4.72	4.39	0.13	3.64–4.93	4
5	**4.06**	4.47	4.44	4.29	0.19	3.07–5.00	2
6	**2.98**	**3.58**	4.17	**3.49**	0.34	1.79–5.00	5
7	**3.20**	**3.64**	4.14	**3.60**	0.31	2.00–5.00	4
8	**3.09**	4.39	4.50	**3.87**	0.26	2.64–5.00	3
9	**3.19**	4.03	4.14	**3.70**	0.39	1.43–5.00	3
10	**3.94**	4.17	4.11	**4.06**	0.33	2.00–5.00	3
11	**3.50**	4.08	4.22	**3.87**	0.28	2.64–4.93	3
12	**2.68**	**3.94**	4.19	**3.47**	0.29	2.21–4.86	5
13	**3.80**	**3.92**	4.14	**3.93**	0.20	3.29–5.00	3
14	4.44	4.78	4.64	4.60	0.14	4.00–5.00	1
15	**4.04**	**2.92**	**3.28**	**3.50**	0.21	2.29–4.21	4

* Values in parentheses indicate the cut-off score within the EQual domain or overall. Bolded values indicate a score that does not reach the cut-off score. SEM: standard error of the mean.

**Table 3:** Variance components from generalizability study associated with EQual ratings of Core EPAs with pharmacist raters (*n* = 9).

Source	Variance	Percent Variance	*n*
EPA (e)	0.138	65.5	1
EQual item (i)	0.007	3.1	14
Rater (r)	0.030	14.2	9
e × i	0.010	4.6	14
e × r	0.025	11.8	9
i × r	0.001	0.6	126
Residual	0.001	0.3	1134

**Table 4:** Summary * of comments and recommendations for EPA revisions (*n* = 9).

Core EPA	Comments	Summary of Recommended Revisions
1	Adequate for describing task and end point Addresses numerous tasks within one EPA	Separate into multiple EPAs
2	Overlaps with another EPA Addresses numerous tasks within one EPA	Separate into multiple EPAs
3	Overlaps with another EPA	Separate into multiple EPAs
4	Overlaps with another EPA Very broad	Separate into multiple EPAs Eliminate
5	Addresses numerous tasks within one EPA	Separate into multiple EPAs
6	Not measurable	Reword for specificity and observability
7	Not restricted to qualified personnel Addresses numerous tasks within one EPA	Eliminate Separate into multiple EPAs Reword to include outcome
8	Lacks context or setting with integration into clinical care Important to the profession	Reword to include context Separate into multiple EPAs
9	Not measurable Use of the word appropriate	Separate into multiple EPAs Reword for measurability
10	Very broad Not specific to the profession of pharmacy	Reword for observability and focus
11	Overlaps with another EPA	Reword for focus
12	Not observable Lacks a clinical outcome	Reword for observability and focus Eliminate
13	Not measurable	Separate into multiple EPAs
14	Singular task Not measurable	
15	Not specific to the profession of pharmacy Not direct component of clinical practice	Eliminate

* Free text responses were summarized by investigators and organized into themes.

## Data Availability

The data presented in this study are available on request from the corresponding author. The data are not publicly available due to privacy restrictions.
